# Novel *BCL11B* truncation variant in a patient with developmental delay, distinctive features, and early craniosynostosis

**DOI:** 10.1038/s41439-022-00220-x

**Published:** 2022-12-05

**Authors:** Kaoru Eto, Osamu Machida, Tomoe Yanagishita, Keiko Shimojima Yamamoto, Kentaro Chiba, Yasuo Aihara, Yuuki Hasegawa, Miho Nagata, Yasuki Ishihara, Yohei Miyashita, Yoshihiro Asano, Satoru Nagata, Toshiyuki Yamamoto

**Affiliations:** 1 Department of Pediatrics, Tokyo Women’s Medical University, Tokyo, Japan; 2 Division of Gene Medicine, Tokyo Women’s Medical University Graduate School of Medicine, Tokyo, Japan; 3 Department of Transfusion Medicine and Cell Processing, Tokyo Women’s Medical University, Tokyo, Japan; 4 Department of Neurosurgery, Tokyo Women’s Medical University, Tokyo, Japan; 5 Department of Plastic and Reconstructive Surgery, Tokyo Women’s Medical University, Tokyo, Japan; 6 Department of Cardiovascular Medicine, Osaka University Graduate School of Medicine, Suita, Japan; 7 Department of Genomic Medicine, National Cerebral and Cardiovascular Center, Suita, Japan; 8 Institute of Medical Genetics, Tokyo Women’s Medical University, Tokyo, Japan

**Keywords:** Neurodevelopmental disorders, Neurodevelopmental disorders

## Abstract

Intellectual developmental disorder with dysmorphic facies, speech delay, and T-cell abnormalities (MIM # 618092) is a congenital disorder derived from pathogenic variants of the B-cell leukemia/lymphoma 11B gene (*BCL11B*). Several variants have been reported to date. Here, through comprehensive genomic analysis, a novel *BCL11B* truncation variant, NM_138576.4(BCL11B_v001): c.2439_2452dup [p.(His818Argfs*31)], was identified in a Japanese male patient with developmental delay, distinctive features, and early craniosynostosis.

B-cell leukemia/lymphoma 11B (*BCL11B*; MIM* 606558) is a lineage-specific, Kruppel-like Cys2-His2 zinc-finger-containing transcriptional regulator that acts bifunctionally as a repressor or activator^1^. In 2016, the first case of human *BCL11B* alteration was reported to have severe combined immunodeficiency (SCID)^2^; the patient also showed intellectual impairment, spastic quadriplegia, and seizures. To date, twenty-seven patients with *BCL11B* alterations have been reported^3^. Recently, we identified a novel *BCL11B* truncation variant in a patient with developmental delay, distinctive features, and early craniosynostosis.

The patient was a 5-year-old Japanese boy who had been delivered via cesarean section (because of breech presentation) with birth weight, birth length, and occipitofrontal circumference (OFC) of 2680 g (10th–25th percentile), 47.8 cm (25th–50th percentile), and 33.5 cm (mean), respectively. The pregnancy was uncomplicated, and there was no asphyxia at birth. Newborn screening for metabolic disorders was negative. There was no history of a compromised infection. His parents were nonconsanguineous, and there was no family history of congenital anomalies. He had sucking difficulties due to his small mouth, although his body weight gain was normal. He achieved head control at 4 months, although he could not maintain his trunk in the prone position and could not roll over at 6 months. His mother brought him to our hospital with suspected gross motor movement delay at 6 months.

At 6 months of age, his body weight, length, and OFC were 7645 g (25th–50th percentile), 68.8 cm (50th–75th percentile), and 43.0 cm (25th–50th percentile), respectively. He showed distinctive features, including prominent forehead, arched eyebrows, midface hypoplasia, thin upper lip, small mouth, long philtrum, retrognathia, and low-set ears (Fig. 1a–d). No abnormal findings were observed in the chest or abdomen. Neurological examination revealed hypotonia with scarf signs. There were no abnormal data in routine blood examinations, including liver function, thyroid function, lactate/pyruvate, and amino acid profiles. Metabolomic analysis of urine also showed normal results. Conventional chromosomal G-banding revealed a normal male karyotype (46,XY). Skull 3D-computed tomography (CT) images at 10 months revealed partial early fusion of the sagittal and lambda sutures (Fig. 1e). Surgical cranioplasty was performed twice, at 12 and 18 months. Brain magnetic resonance imaging (MRI) at 11 months showed a slightly reduced volume of the cerebrum but no abnormal signals (Fig. 1f, g).

**Fig. 1:**
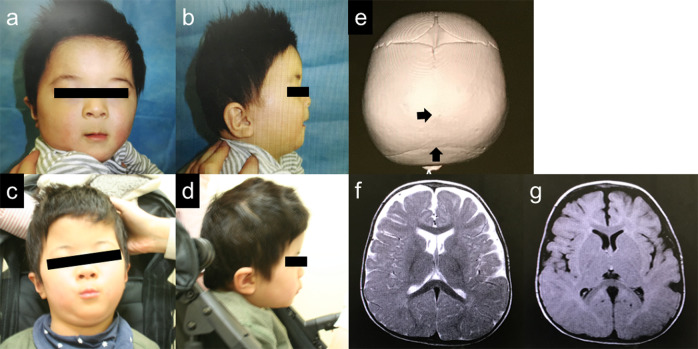
Clinical information of the patient. Facial appearance of the patient at 6 months of age (**a**, **b**) and 5 years of age (**c**, **d**), suggesting deformity of the skull, forehead protrusion, arched eyebrows, flat nose base, thin upper lip, small mouth, long philtrum, retrognathia and low-set ears. **e** 3-dimensional CT image of the skull at 10 months of age shows partial early fusion of the sagittal and lambda sutures (arrows). T1-(**f**) and T2-(**g**)-weighted brain MRI at 11 months of age showed no apparent abnormalities.

His motor development was delayed, with rolling over occurring at 8 months, sitting without support at 10 months, crawling at 11 months, and walking alone at 28 months. In addition, his language development was severely delayed, with no speaking or mimicking. Furthermore, he exhibited autistic behaviors at three years of age. He started baby food at 5 months of age, although he had difficulty feeding using a spoon due to sensitivity around the mouth. Thus, he consumed baby food dissolved with formula using a baby bottle. He was allergic to eggs, milk, wheat, and soy. He started oral rehabilitation and was gradually able to eat food with a spoon at 3 years of age. He had no history of recurrent infection, and his laboratory data, including immunoglobin; CD4/CD8 ratio; and CD3, CD10, CD19, and CD20 profiles, were normal.

For precise diagnosis, this patient was enrolled in the research project “Initiative on Rare and Undiagnosed Disorders (IRUD)”, which was performed in accordance with the Declaration of Helsinki and approved by the ethics committee of our institution^4^. After obtaining informed consent from the family, blood samples were collected from the patient and his parents. Genomic DNA was extracted from the peripheral blood samples following a standard protocol, and exome sequencing was performed as described previously using trio samples, including parental samples, at 3 years and 11 months of age^5^. The results showed a de novo heterozygous variant of NM_138576.4(BCL11B_v001):c.2439_2452dup [p.(His818Argfs*31)]. Standard Sanger sequencing confirmed this finding (Fig. 2a). The identified variant has not been reported previously and is not registered in any database. The clinical information of this patient is summarized in Table 1 together with previous reports^2,3,6–15^.

**Fig. 2:**
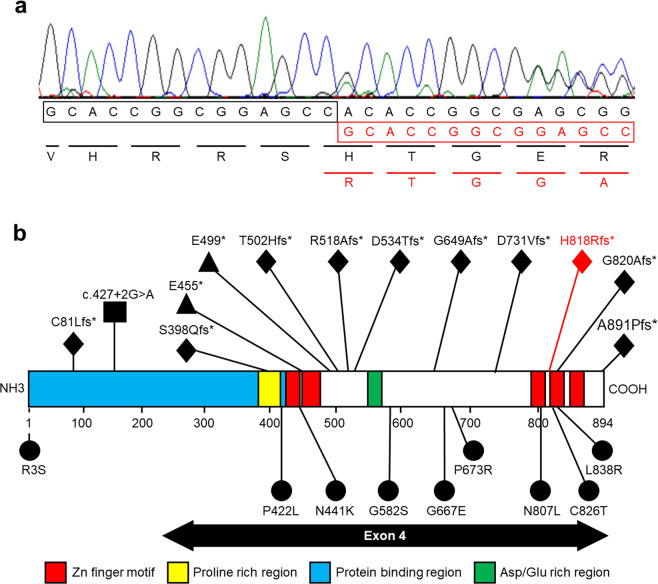
Results of genetic analysis. **a** Sanger sequencing indicates overlapping electropherograms due to the 14-bp duplication. **b** Locations of the *BCL11B* variants are depicted on the primary structure (modified data from Prasad et al., 2020). The positions of the previously reported *BCL11B* variants are shown in black. The variant reported in this study is shown in red (in the 4th zinc-finger motif). Most of the variants are located on exon 4. Circles, rhombuses, triangles, and rectangles indicate missense, truncation, nonsense, and splicing mutations, respectively. NH3 N-terminus, COOH C-terminus.

**Table 1:** Comparison of the clinical features of the reported patients with *BCL11B* alterations.

Authors	Present patient	Punwani et al.	Lessel et al.	Goos et al.	Qiao	Homma et al.	Prasad et al.	Yang et al.	Gaillard et al.	Lu et al.	Alfei et al.	Harrer et al.	Che et al.
Published year		2016	2018	2019	2019	2019	2020	2020	2021	2021	2021	2022	2022
Gender (number of cases)	M (1)	M (1)	M (6), F (4)^a^	M (1)	F (1)	M (1)	F (2)	F (1)	M (2), F (2)	F (1)	M (1)	F (1)	M (2), F (2)
Cognitive/motor development													
Intellectual disability	+	+	12/12	-	+	+	1/2	+	0/4	+	+	+	+
Speech impairment	+	+	12/12	NA	+	+	2/2	+	2/4	NA	NA	+	NA
Delay in motor development	+	+	11/12	NA	+	+	2/2	+	2/4	NA	+	+	NA
Autistic features	+	-	4/12	NA	-	NA	NA	-	NA	NA	NA	NA	NA
Dysmorphic features													
Myopathic face appearance	-	-	6/12	-	-	NA	1/2	NA	1/4	NA	NA	NA	NA
Thin eyebrows	+	+	5/12	+	+	NA	2/2	+	1/4	NA	+	NA	NA
Small palpebral fissures	+	+	7/12	+		NA	2/2	NA	0/4	NA	+	NA	NA
Hypertelorism	±	+	7/12	+	+	NA	2/2	+	1/4	NA	+	NA	NA
Prominent nose	+	-	9/12	-	+	NA	1/2	-	2/4	NA	NA	NA	NA
Long philtrum	+	+	9/12	+	+	NA	2/2	+	2/4	NA	+	NA	NA
Thin upper lip vermilion	+	+	11/12	+	+	NA	2/2	+	3/4	NA	NA	NA	NA
Craniosynostosis	+	-	-	+	-	NA	0/2	NA	4/4	NA	NA	NA	NA
Others													
Refractive error	-	-	+	NA	-	NA	NA	NA	3/4	NA	+	NA	NA
Dental anomalies	-	+	5/12	NA	-	NA	1/2	NA	0/4	NA	+	NA	NA
Feeding problems	+	-	3/12	-	-	NA	2/2	NA	1/4	NA	+	NA	NA
Epilepsy	-	NA	NA	+	NA	NA	NA	NA	NA	NA	-	NA	NA
Brain MRI abnormalities	-	+ (^b^)	NA	-	-	NA	1/2 (^c^)	+	NA	-	+	+	NA
Immune system function													
Immune response	-	+	5/12	NA	^d^	+	0/2	+	1/4	+	+	NA	NA
Allergy/asthma	+	+	7/12	NA	NA	NA	0/2	NA	1/4	+	NA	NA	NA
*BCL11B* alterations	p.H818Rfs*31	p.N441K	p.C81Lfs*76 p.Y455* p.E499* p.T502Hfs*15 p.R518Afs*45 p.D534Tfs*29 p.G649Afs*67 p.N807L p.G820Afs*27 p.A891Pfs*106	p.R3S	p.D731Vfs*150	p.C81Lfs*76	p.N807L p.G649Afs*67	p.S398Qfs*117	p.P422L p.G582S p.G667E p.P673R	p.C826T	p.N441K	p.L838R	c.427 + 2 G > A

This table is modified from that reported by Lessel et al. (2018)^3^.

^a^Only the case with nucleotide alterations are included.

^b^Agenesis of the corpus callosum.

^c^Dysgenesis of corpus callosum/enlargement of lateral ventricles.

^d^Episodes of frequent infections.

BCL11B plays an essential role in the development of the nervous, immune, and cardiovascular systems and is also involved in skin, dental, and cranial development^16^. In murine models, biallelic loss of Bcl11b leads to defects in the development of the central nervous system^17,18^, epidermis^19^, and teeth^3,20^, as well as in the development and maintenance of the T-cell lineage^21^, resulting in perinatal lethality. The role of Bcl11b in craniofacial skeleton formation has also been reported in murine models^22,23^; however, it is less common in humans.

The first reported case of a *BCL11B* variant, p.N441K, exhibited SCID associated with neurological features^2^. The identified variant is located in the zinc-finger C2H2-type domain. Thus, a dominant-negative effect on the DNA-binding structural interface was suggested. A subsequent report included 27 additional patients with *BCL11B* alterations, including our patient (Table 1). In total, 21 variants have been reported, including frameshift variants (9), missense variants (9), nonsense variants (2), and a splicing variant (1) (Fig. 2b). Patients harboring missense variants may show the most severe clinical findings, which also supports the mechanism of dominant-negative effects. However, variants leading to protein truncation (frameshift and nonsense) were shared by more than half of patients. Truncation variants are predicted to activate nonsense-mediated mRNA decay, resulting in haploinsufficiency. However, 10 truncation variants are located in the last exon (exon 4) and are thus predicted to escape nonsense-mediated mRNA decay, probably resulting in a protein with loss of C-terminal DNA-binding zinc-finger domains^3^.

*BCL11B* is located on human chromosome 14p32.2 and consists of four exons, and it encodes five zinc-finger C2H2-type domains. Three of them were located in the C-terminal region of the last exon (Fig. 2b). BCL11B has an amino acid length of 894, and over half of the amino acids are encoded by exon 4 (Fig. 2b). Therefore, most truncation variants are predicted to cause the loss of the last C-terminal DNA-binding zinc-finger domains.

In this study, we identified a unique 14-bp tandem duplication in exon 4, which was predicted to be involved in protein truncation, as discussed above. This patient showed severe developmental delay, autistic features, and distinctive findings of craniosynostosis, which was repaired by cranioplasty in early infancy.

Craniosynostosis is a disorder of skull formation caused by premature ossification of cranial sutures, occurring in ~1 in 2250 births^6^. Although the first two reports of *BCL11B* alterations showed no findings of craniosynostosis, five patients have been reported to show craniosynostosis in association with *BCL11B* alterations, suggesting a relatively less common manifestation in humans^6,11^.

Genetic alterations have been identified in approximately a quarter of craniosynostosis cases. The most frequent activating mutations are in genes coding for FGF receptors^24,25^, as well as for other genes, including the twist basic helix-loop-helix transcription factor 1 gene (*TWIST1*) and the ephrin-B2 gene (*EFNB*). Owing to advances in comprehensive genetic testing, several *BCL11B* alterations have recently been reported in children with craniodiaphyseal abnormalities. Bcl11b regulates FGF-dependent signaling pathways. Analysis of Bcl11b-deficient mice and neural crest-specific inactivation (Bcl11b ncc–/–) mice revealed its role in craniofacial development and maintenance of suture patency^22,23^. Furthermore, Bcl11b is highly expressed in the mesenchyme of the murine craniofacial skeleton during embryonic development^22,23^.

The variants of the five reported cases of craniosynostosis were missense variants (p.Arg3Ser, p.Gly667Glu, p.Gly582Ser, p.Pro673Arg, and p.Pro422Leu), and four of the five variants were located in exon 4^6,11^. Gaillard et al.^11^ hypothesized that these missense variants may either have a dominant-negative effect or induce new target genes, thereby causing a more severe phenotype in patients with these missense variants. These findings indicate that truncation variants usually cause loss of function, but the variant identified in this study may have a dominant-negative effect.

## Data Availability

The relevant data from this Data Report are hosted at the Human Genome Variation Database at 10.6084/m9.figshare.hgv.3255.
